# Dataset on physical properties of raw and roasted cashew nuts

**DOI:** 10.1016/j.dib.2020.106514

**Published:** 2020-11-06

**Authors:** Oluwaseun Kilanko, Sunday J Ojolo, Richard O Leramo, Titus A Ilori, Sunday O Oyedepo, Phillip O Babalola, Ojo SI Fayomi, Patrick N Onwordi, Edidiong Ufot, Akaninyene Ekwere

**Affiliations:** a Department of Mechanical Engineering, Covenant University, Nigeria; b Mechanical Engineering Department, University of Lagos, PMB 56, Akoka, Yaba, Lagos State, Nigeria; c Department of Agriculture Engineering Programme, Federal College of Agriculture, Ibadan, Nigeria

**Keywords:** Cashew, Physical properties, Nut-size, Hot-oil, Roasting, Density, Sphericity

## Abstract

Cashew nut is one of the topmost edible crops in the world. However, one of the challenges of this crop is processing. Designing an equipment for the processing of cashew nut requires the knowledge of its physical properties data. The dataset in this article contained the physical properties of raw and roasted cashew nuts. The physical properties include length, width, thickness, geometric mean diameter, sphericity, true density, bulk density, porosity and mass of cashew nut. Two experiments were performed. In one experiment, raw cashew nut was roasted in groundnut oil. In the second experiment, raw cashew nut was roasted in palm-kernel oil. The physical properties of the nuts were measured before and after roasting in hot oil. The data were subjected to a paired sample t-test analysis to determine the level of significant difference. The data of the cashew nut graded with machine and sorted with hand manually were compared. The data provided in this article will be useful in designing various types of equipment for grading, separating and cleaning cashew nut. It will also be useful in the design of storage structures and processing machines.

## Specifications Table

**Table:** 

Subject Area	Engineering
More Specific Subject area	Agricultural Engineering and Mechanical Engineering
Type of Data	Tables and Figures
How Data was Acquired	The length, width and thickness of the cashew nut were measured using Carrera Precision Vernier Digital Caliper which measure to an accuracy of 0.01 mm. The geometric mean diameter and sphericity were calculated from these measured values. The mass of the nut was measured using Ohaus digital weighing balance that measured to an accuracy of 0.0001 g. The true density of the nut was determined using water displacement method which is the ratio of the mass of the nut to the volume of water displaced in a measuring cylinder when the nut was immersed in water. The bulk density of the cashew nut was determined by the ratio of the weight of the cashew nuts contained in a cylindrical container to the volume of the container. Porosity of the cashew nut was determined from the values of true density and bulk density of the nut. A wire mesh basket was made and divided into 25 compartments for the roasting of the nuts in hot-oil. This was done to track each of the nut in order to obtain a set of data for the same nut before and after roasting in hot-oil.
Data Format	Raw and analysed
Parameters for data collection	The raw cashew nuts were roasted at moisture content of 7.00% w.b. in two hot-oil roasting media (groundnut oil and palm-kernel oil) at roasting temperature between 190 °C–210 °C and roasting time of 90 s.
Description of data collection	The physical properties of the cashew nut were determined before and after roasting in hot-oil media. The two sets of data were compared statistically to determine whether roasting cashew nut in hot-oil medium has significant effect (at *p* < 0.05) on its physical properties
Data source location	Department of Mechanical Engineering, Covenant University, Ota, Nigeria
Data Accessibility	Data are available within this article
Related Research Article	[1] O. Kilanko, S.J. Ojolo, A.O Inegbenebor, T.A. Ilori, R.O. Leramo, P.O. Babalola, P.N. Onwordi. Effect of Pre-Shelling Treatment on Physical and Mechanical Properties of Cashew Nut. IOP Conference Series: Materials Science and Engineering 413 (1) (2018), 012038. 10.1088/1757-899X/413/1/012038

## Value of the Data

• The data provides relevant information needed for the design of storage facilities and engineering equipment for the processing of cashew nut.
• Researchers that are interested in designing machineries for grading, separating, cleaning shelling of the cashew nuts may use this data for their design parameters.
• The data is comprehensive, containing 9 physical properties of cashew nut (length, width, thickness, geometric mean diameter, sphericity, true density, bulk density, porosity and mass).
• The data can be used to determine the effect of hot-oil roasting media on the physical properties of cashew nuts.

## 1 Data Description

Cashew nuts were procured from two different sources in Nigeria. These cashew nuts were sorted into three categories; large size, medium size and small size. Nine (9) physical properties of the raw cashew nuts were measured before roasting in hot-oil. The same set of properties were measured after roasting the nuts. These properties are length, width, thickness, geometric mean diameter, sphericity, true density, bulk density, porosity and mass. The first experiment was carried out on samples of cashew nut obtained from a location in Nigeria. The nut was roasted in palm-kernel oil. The second experiment was performed on another samples of cashew nut obtained from another location in Nigeria. These nuts were roasted in groundnut oil. The set of data generated from these experiments were compared statistically to determine whether the roasting in hot-oil and the medium of roasting have significant effects on the physical properties of the cashew nuts. Supplementary Tables 1–4 showed data obtained from the first and second experiments. Supplementary Tables 1 and 3 presented the length, width, thickness, geometric mean diameter and sphericity of the raw cashew nut and cashew nut roasted in groundnut oil and palm-kernel oil. Supplementary Tables 2 and 4 presented the data on true density, bulk density, porosity and mass of the raw and roasted cashew nut for the two experiments. Table 1 showed the descriptive statistics of physical properties of cashew nuts before and after roasting for Groundnut oil. This table showed the number of counts, the average value and the standard deviation of the physical properties of the cashew nuts. The descriptive statistics of physical properties of cashew nuts before and after roasting in palm-kernel oil was presented in study done by Kilanko et al [1].

**Table 1:** Descriptive statistics of physical properties of cashew nuts before and after roasting in Groundnut oil.

		Large		Medium		Small	
Properties	N	Raw	Roasted	Raw	Roasted	Raw	Roasted
Length (mm)	300	34.78 (2.5)	36.32 (2.5)	31.08 (1.7)	32.75 (1.7)	29.13 (2.2)	30.55 (2.1)
Width (mm)	300	26.26 (1.9)	28.07 (2.0)	23.70 (1.0)	25.68 (1.2)	22.18 (1.6)	23.92 (1.6)
Thickness (mm)	300	18.15 (1.8)	19.63 (1.7)	17.17 (1.6)	18.67 (1.4)	16.22 (1.4)	17.52 (1.5)
GMD (mm)	300	25.46 (1.6)	27.12 (1.6)	23.27 (1.0)	25.01 (1.0)	21.86 (1.3)	23.37 (1.3)
Sphericity (%)	300	73.32 (3.4)	74.78 (3.2)	74.99 (3.7)	76.50 (3.4)	75.23 (4.21)	76.65 (3.9)
True density (g/cm^3^)	60	1.022 (0.08)	0.777 (0.11)	1.08 (0.08)	0.736 (0.1)	1.06 (0.07)	0.767 (0.1)
Bulk density (g/cm^3^)	60	0.55 (0.02)	0.423 (0.03)	0.545 (0.02)	0.403 (0.03)	0.555 (0.01)	0.426 (0.05)
Porosity (%)	60	45.87 (4.84)	44.87 (6.4)	49.28 (4.20)	44.57 (6.7)	47.36 (73.56)	43.83 (6.0)
Mass of the nut (g)	60	7.47 (1.59)	6.98 (1.7)	5.68 (0.66)	5.00 (0.8)	4.80 (0.79)	4.28 (0.8)

## 2 Experimental Design, Materials and Methods

Shelling is one of major challenges of cashew nut processing. However, before shelling, the cashew nut is first pre-treated. One of the pre-treatments of the nut is roasting which are the experiments performed in this paper. Two experiments were performed in this study. The first experiment was performed with raw cashew nuts sourced at Ilorin in Kwara State of Nigeria. In this experiment, groundnut oil was used as roasting medium. The second experiment was performed with raw cashew nuts sourced at Iwo in Osun State of Nigeria. In this experiment, palm-kernel oil was used as roasting medium. Because of shortage or non-availability of CNSL, groundnut cooking oil and palm-kernel oil were used as alternative roasting media [1], [2], [3]. The raw cashew nuts were roasted at a moisture content level of 7.00% w.b. The two batches of procured cashew nuts were sorted into three categories of different nut sizes (large, medium, and small). The cashew nut used in the first experiment was sorted by an India cashew nut grading machine. The cashew nut used in the second experiment was sorted manually by their axial dimensions according to Balasubramania [4]. For each experiment, 300 nuts were selected from large, medium and small cashew nuts, making a total of 900 cashew nuts. For the two experiments, a total of 1800 cashew nuts were selected and their physical properties (length, width, thickness, geometric mean diameter and sphericity) were measured before and after roasting. However, out of the 300 cashew nuts selected per nut size, 60 of them were selected and their true density, bulk density, porosity and mass were determined. This made a total of 180 nuts per experiment and a total of 360 nuts for the two experiments. For roasting in bath of hot-oil, 25 nuts were roasted at a time. A wire mesh basket was made and divided into 25 sections. In order to track each nut before and after roasting, these nuts were labelled and put into the basket [1]. The wire mesh basket was dipped into hot-oil for 90 seconds at temperature range between 190 °C–210 °C. For each experiment, the roasting was done repeatedly until the 900 cashew nuts were roasted. The nuts were allowed to cool naturally after roasting [4], [5].

The measurement of the physical properties of the cashew nut was done before and after roasting. The three axial dimensions of the nut (length (a), width (b) and thickness (c)) were measured using digital Vernier caliper to an accuracy of 0.01 mm. The geometric mean diameter and sphericity of the nuts were calculated from the axial dimensions. The mass of the nut was measured using Ohaus digital weighing balance that measured to an accuracy of 0.0001 g. The true density of the nut was determined using water displacement method which is the ratio of the mass of the nut to the volume of water displaced in a measuring cylinder when the nut was immersed in water. The bulk density of the cashew nut was determined by the ratio of the weight of the cashew nuts contained in a cylindrical container to the volume of the container. Porosity of the cashew nut was determined from the values of true density and bulk density of the nut. The equations for the calculated parameters were given by Kilanko et al [1]*,* Ogunsina and Bamgboye [6].

The statistical analysis was performed on the data obtained from the experiments. The data of the properties of raw cashew nut was compared statistically with the roasted cashew nut properties in each experiment (Table 2) to determine whether heat treatment has significant effect on the properties. The data of the properties of raw and roasted cashew nut was compared statistically (Table 3) between the two experiments (cashew nuts graded with machine and sorted with hand manually) to determine whether there is significant difference on the properties. The statistical analysis used on these data was paired t-test statistics. The t-test statistics and the degree of freedom were computed using the following equations according to Zhu et al [7]:

(1) $$\;\;t=\frac{m}{s\;/\;\sqrt{n}}$$

(2) df=n−1

m = mean of the difference (d)
s = standard deviation of the difference (d)
n = sample size (i.e. size of d)
df = degree of freedom

**Table 2:** Effect of heat-treatment on Physical properties of cashew nuts.

		t-statistics					
		Groundnut oil			Palm-kernel oil		
Properties	df	Large	Medium	Small	Large	Medium	Small
Length	299	−30.05*	−34.70*	−16.52*	−12.65*	−20.67*	−35.08*
Width	299	−23.08*	−40.19*	−28.67*	−16.49*	−22.08*	−21.20*
Thickness	299	−28.83*	−27.26*	−23.99*	−29.36*	−30.07*	−31.66*
GMD	299	−40.75*	−52.96*	−31.30*	−30.16*	−40.60*	−42.93*
Sphericity	299	−12.92*	−13.96*	−7.14*	−16.94*	−16.54*	−6.55*
True density	59	19.89*	25.65*	18.13*	23.47*	24.18*	32.36*
Bulk density	59	29.41*	42.47*	19.72*	46.02*	55.82*	59.15*
Porosity	59	1.37	6.36*	4.96*	1.75	2.25*	−0.11
Mass	59	9.90*	14.12*	10.12*	14.05*	9.41*	14.55*

⁎ t-statistics are significant at 5% probability level.

**Table 3:** Comparison between the raw and roasted cashew nuts graded with machine and sorted with hand.

		t-statistics					
		Raw Cashew nuts			Roasted Cashew nuts		
Properties	df	Large	Medium	Small	Large	Medium	Small
Length	299	−11.77*	−2.09*	−24.10*	−15.46*	−8.47*	−22.96*
Width	299	−8.08*	−2.66*	−16.07*	−12.42*	−13.89*	−21.75*
Thickness	299	−6.77*	−7.60*	−14.87*	−6.65*	−8.61*	−12.54*
GMD	299	−10.55*	−7.54*	−23.13*	−13.69*	−14.52*	−22.64*
Sphericity	299	1.39	−4.00*	5.80*	3.62*	−3.67*	4.22*
True density	59	−1.76	−2.56*	0.47	−2.81*	−0.20	−4.17*
Bulk density	59	24.49*	27.05*	24.85*	10.85*	13.49*	−3.59*
Porosity	59	−11.36*	−11.81*	−6.91*	−7.41*	−6.35*	−2.13*
Mass	59	1.40	1.05	−6.88*	−0.90	0.45	−6.97*

⁎ t-statistics are significant at 5% probability level.

**Fig. 1:**
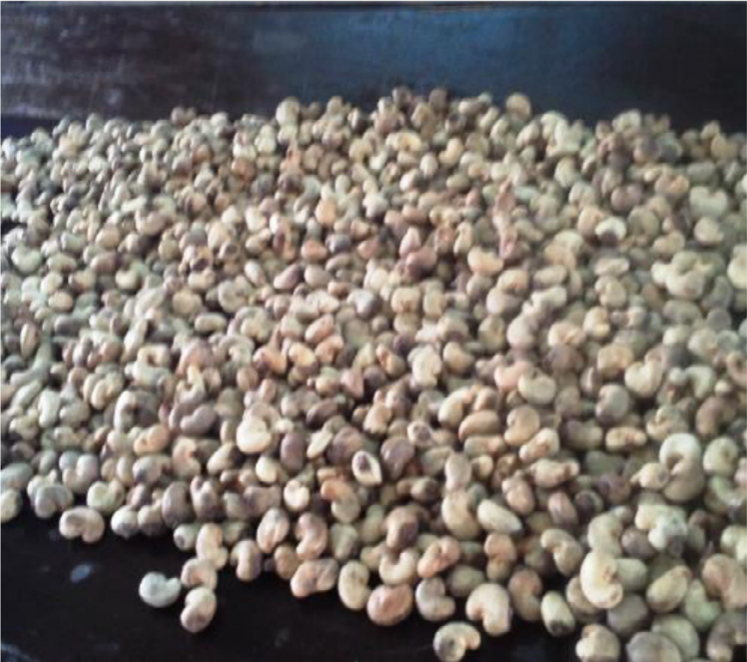
Raw cashew nut spread out.

**Fig. 2:**
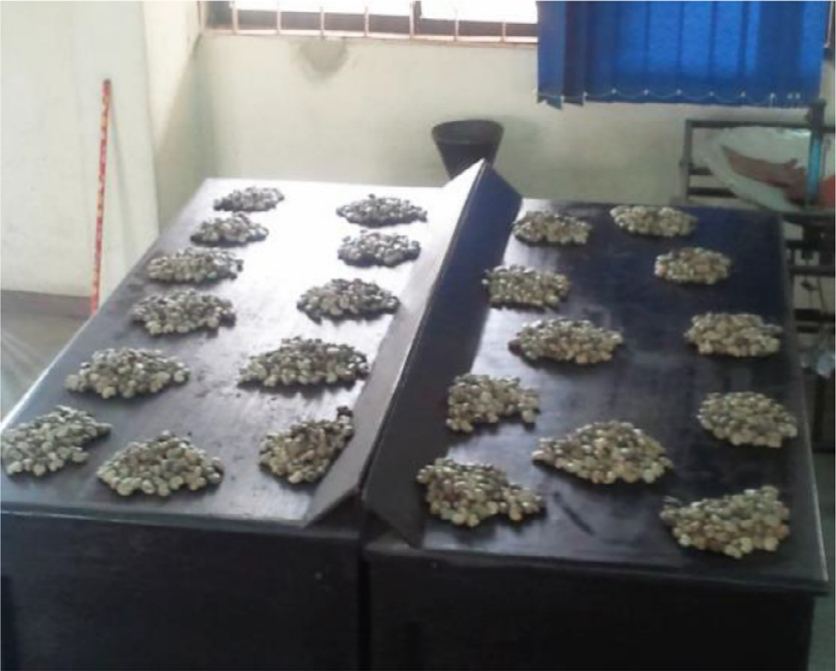
Raw cashew nut in batches.

The t-statistics was calculated at 95% confidence interval.

In Table 2, the data indicates the heat-treatment has significant effect (at P < 0.05) on the physical properties of cashew nut roasted in the two oils.

**Fig. 3:**
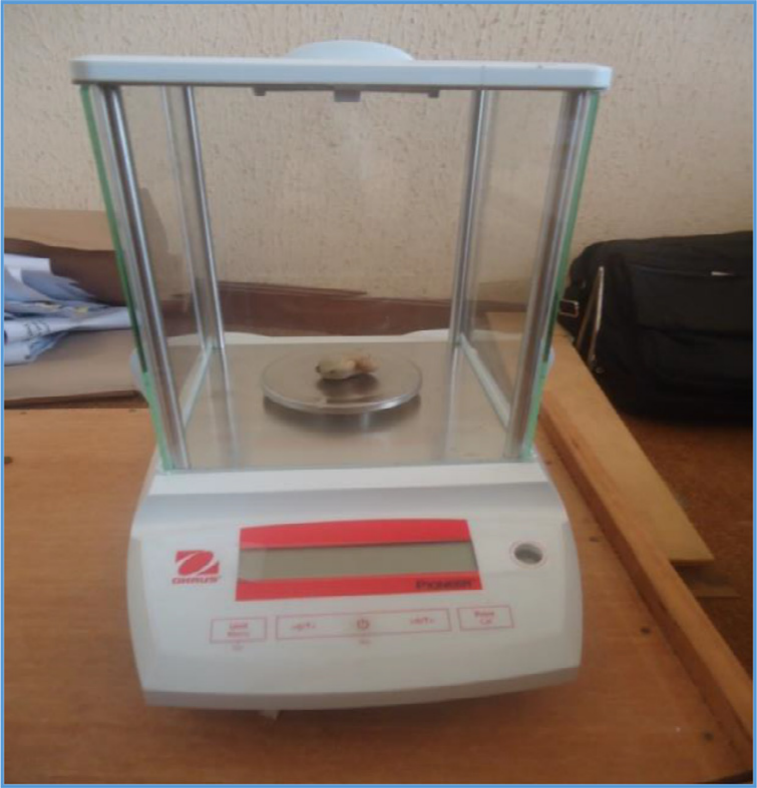
Electronic Weighing Balance.

**Fig. 4:**
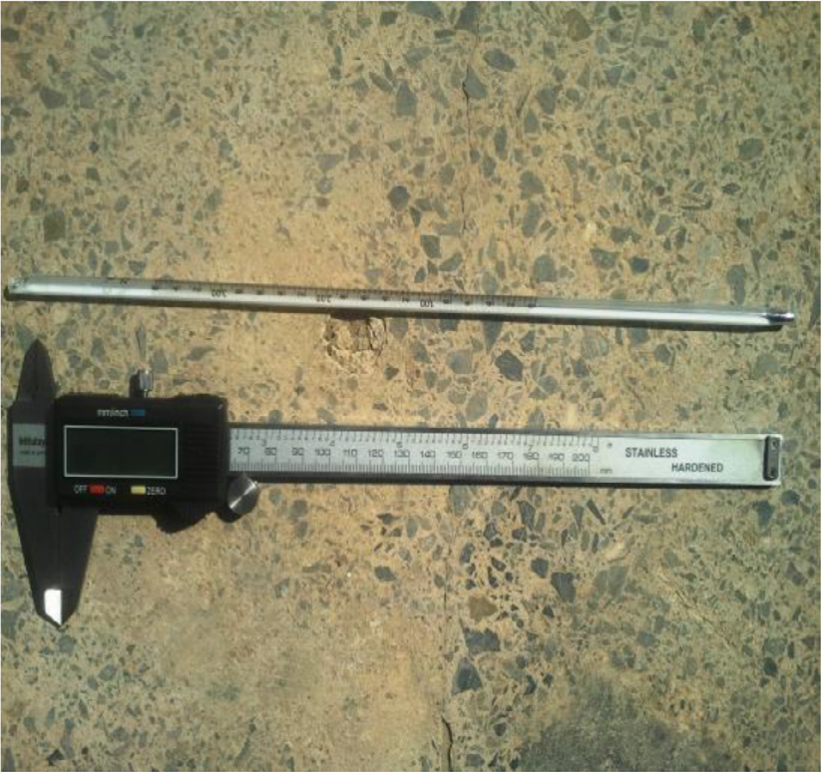
Digital Venier caliper and Thermometer.

**Fig. 5:**
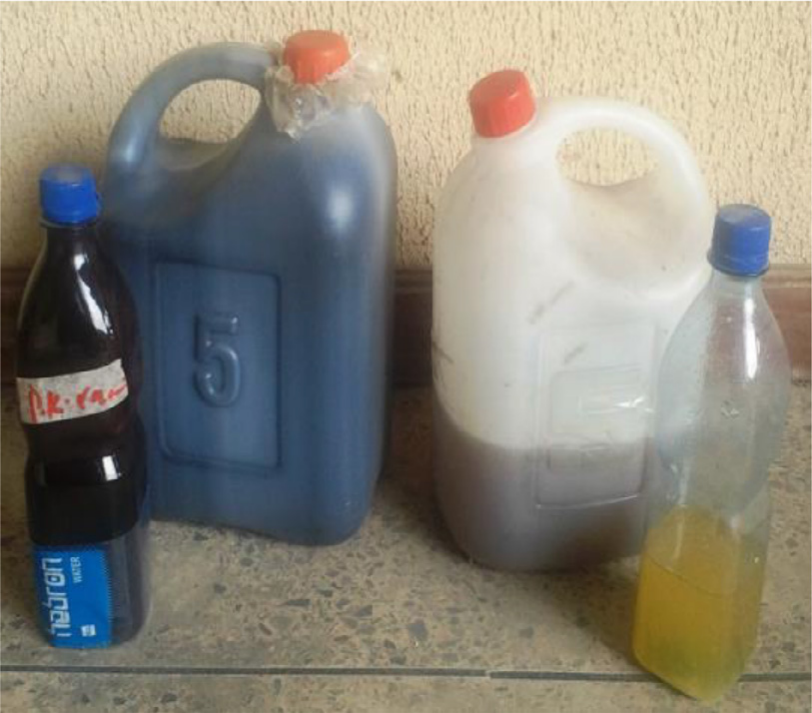
Groundnut oil and Palm Kernel oil.

**Fig. 6:**
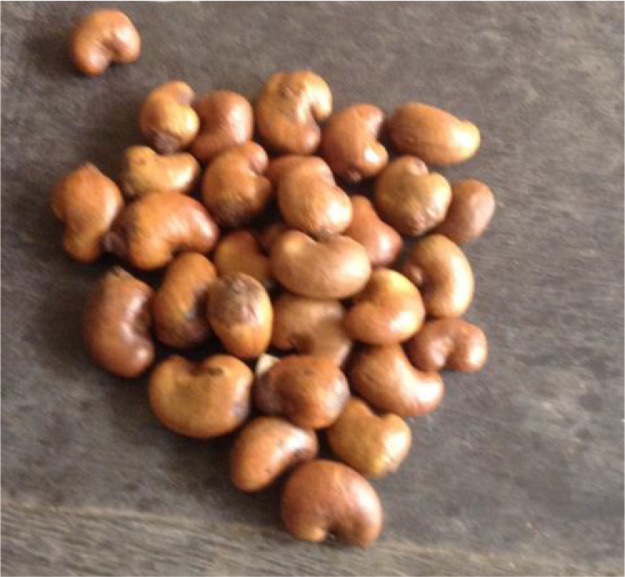
Roasted cashew nuts.

**Fig. 7:**
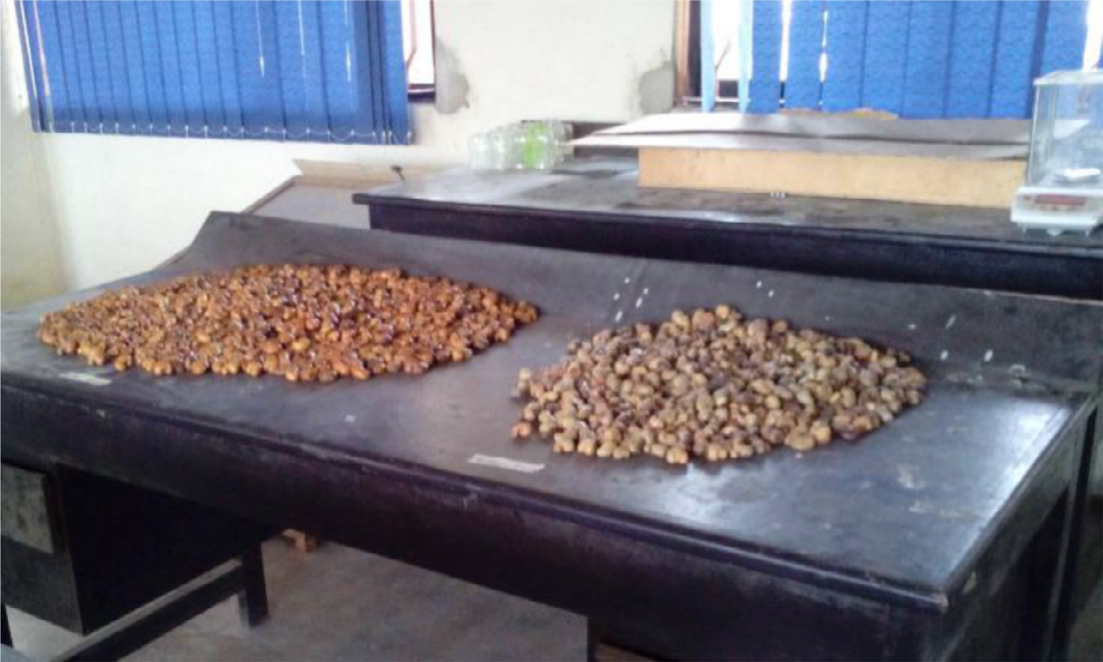
Roasted and raw cashew nuts.

The figures below (Fig. 1, Fig. 2, Fig. 3, Fig. 4, Fig. 5, Fig. 6, Fig. 7) showed some pictures taken while the experiments were being performed.

## CRediT Author Statement

**Oluwaseun Kilanko:** Conceptulization, Methodology, Writing – Original Draft. **Sunday J. Ojolo:** Methodology, Supervision. **Richard O. Leramo:** Methodolgy, Writing – Review and Editing. **Titus Ilori:** Methodology, Supervision, Resources. **Sunday O. Oyedepo:** Data analysis. **Phillip O. Babalola:** Supervision. **Ojo S.I. Fayomi:** Methodology, Writing – Review and Editing. **Patrick N Onwordi:** Investigation, Resources. **Edidiong Ufot:** Investigation, Writing – Review and Editing. **Akaninyene Ekwere:** Investigation, Writing – Review and Editing.

## Declaration of Competing Interest

None

## Appendix Supplementary materials

Image, application 1

## Footnotes

Supplementary material associated with this article can be found in the online version at doi:10.1016/j.dib.2020.106514.
